# Plastic and Heritable Variation in Shell Thickness of the Intertidal Gastropod *Nucella lapillus* Associated with Risks of Crab Predation and Wave Action, and Sexual Maturation

**DOI:** 10.1371/journal.pone.0052134

**Published:** 2012-12-13

**Authors:** Sonia Pascoal, Gary Carvalho, Simon Creer, Sonia Mendo, Roger Hughes

**Affiliations:** 1 Molecular Ecology and Fisheries Genetics Laboratory, School of Biological Sciences, Environment Centre Wales, Bangor University, Gwynedd, United Kingdom; 2 CESAM – Centre for Environmental and Marine Studies & Department of Biology, University of Aveiro, Campus Universitário de Santiago, Aveiro, Portugal; Sheffield University, United States of America

## Abstract

The intertidal snail *Nucella lapillus* generally has thicker shells at sites sheltered from wave action, where crabs are abundant and pose a high risk of predation, than at exposed sites where crabs are rare. We studied two populations showing the opposite trend. We reciprocally transplanted snails between field sites and measured shell length, width and lip thickness of those recaptured 12 months later. Snails transplanted to the sheltered site grew larger than sheltered-site residents, which in turn grew larger than transplants to the exposed site. Relative shell-lip thickness was greater in residents at the exposed site than at the sheltered site. Transplants from shelter to exposure developed relatively thicker shells than their controls and relatively thinner shells from exposure to shelter. Progeny of the two populations were reared for 12 months in a common garden experiment presenting effluent from crabs feeding on broken conspecifics as the treatment and fresh sea-water as the control. The crab-effluent treatment decreased foraging activity, concomitantly reducing cumulative somatic growth and reproductive output. Juveniles receiving crab-effluent grew slower in shell length while developing relatively thicker shell lips than controls, the level of response being similar between lineages. F_2_ progeny of the exposed-site lineage showed similar trends to the F_1_s; sheltered-site F_2_s were too few for statistical analysis. At sexual maturity, shell-lip thickness was greater in snails receiving crab-effluent than in controls, indicating plasticity, but was also greater in the exposed-site than in the sheltered-site lineage, indicating heritable variation, probably in degree of sexual thickening of the shell lip. Results corroborate hypotheses that ‘defensive’ shell thickening is a passive consequence of starvation and that heritable and plastic control of defensive shell morphology act synergistically. Shell thickening of juveniles was similar between lineages, contrary to hypotheses predicting differential strengths of plasticity in populations from low- or high-risk habitats.

## Introduction

It is widely reported that certain rocky shore gastropods develop thicker-walled shells at sites sheltered from wave action, where crabs are often abundant and pose a high risk of predation, than at exposed sites where crabs tend to be scarce (e.g. [1]–[3]). Shell-wall thickness, often measured at the aperture lip (e.g. [4]), has been experimentally demonstrated to increase in response to olfactory cues associated with risk of crab predation [5]–[7] possibly directly [8], [9], or indirectly through starvation resulting from inhibited foraging [10], [11]. Not only shell-wall thickness but also shell shape is known to influence vulnerability to crab predation [1], [12]–[15]. Whereas in some cases resistance to crab attack is gained by reduced aperture area, often correlated with narrowing of the aperture and elongation of the shell spiral [1], [8], [12], [13], [15]–[18], in other cases resistance is increased by globosity that hinders grip on the shell-body whorl [19], [20]. Because crabs tend to be more numerous at sites sheltered from wave action, or forage throughout the longer periods of tidal immersion at lower shore levels, defensive shell morphology tends to be more pronounced in such environments. At sites exposed to wave action or at higher shore levels, adaptive shell morphology increases resistance to dislodgement or to physiological stressors while trading-off defensive attributes [1], [3], [15]. Shell morphology has been shown to be under both heritable and plastic control, which may act synergistically [17], [18], [20], [21]. In some cases, induced defensive shell morphology is more pronounced in populations from crab-infested habitats than in those from crab-free habitats [21], but the opposite may be true of other cases [8]. Furthermore in certain taxa, sexual maturation may involve thickening of the aperture lip [22], confounded with any relationship between risk of crab predation and lip thickness of the adult shell. Evidently, the induction of defensive shell morphology involves a complex of factors requiring investigation over a range of populations within and among taxa in order to reach better understanding.

Accordingly, we combined a reciprocal transplant experiment in the field with common garden experiments deploying laboratory-hatched progeny to examine plastic and heritable components of variation in shell morphology in two populations of *Nucella lapillus* (L.) that contravene the general trend by having thicker shells at a site exposed to heavy wave action and free of crabs than at a more sheltered site infested with crabs. In contrast to shell thickness, shell shape in the two populations follows the normal trend for *N. lapillus* in which shells at exposed sites have relatively shorter spires and larger, wider apertures than shells at sheltered sites.

The reciprocal transplant and common garden experiments were designed to examine the roles of inheritance and plasticity on adaptive shell morphology and to yield samples for studying associated gene expression. Because of the large volume of data, effects of reciprocal transplantation and common garden conditions lacking wave action or crab-effluent on shell shape are presented elsewhere [18]. Here, we examine the effects of crab-effluent, presumed to signal predation risk, on thickness and shape of the shell. While focussing on the common garden experiment, we also present data on shell thickness from the reciprocal transplant experiment to aid interpretation of results.

## Materials and Methods

Source populations of *N. lapillus* were obtained from two sites in North Wales, U.K. (for a map see Pascoal et al. [18]). One site, Cable Bay (53° 18.357′ N, 04° 08.293′ W) is exposed to strong wave action generated by prevailing south-westerly winds from the Southwestern Approaches and across the Irish Sea, while the other, Llanfairfechan (53° 15.456′ N, 03° 58.085′ W), is sheltered in the lee of the prevailing winds. Adults were collected in February 2008 and maintained in site-dedicated 126-litre aquaria supplied with running seawater closely tracking ambient outdoor temperature. Barnacles were supplied as prey and replenished as needed. Spawning aggregations were formed and egg masses deposited on the walls of the aquaria. Once hatched juveniles (F_1_s) had grown to a shell length of 8–12 mm (August 2008), they were colour-coded with a waterproof pen according to lineage (exposed or sheltered).

### Reciprocal transplant experiment

**Figure 1:**
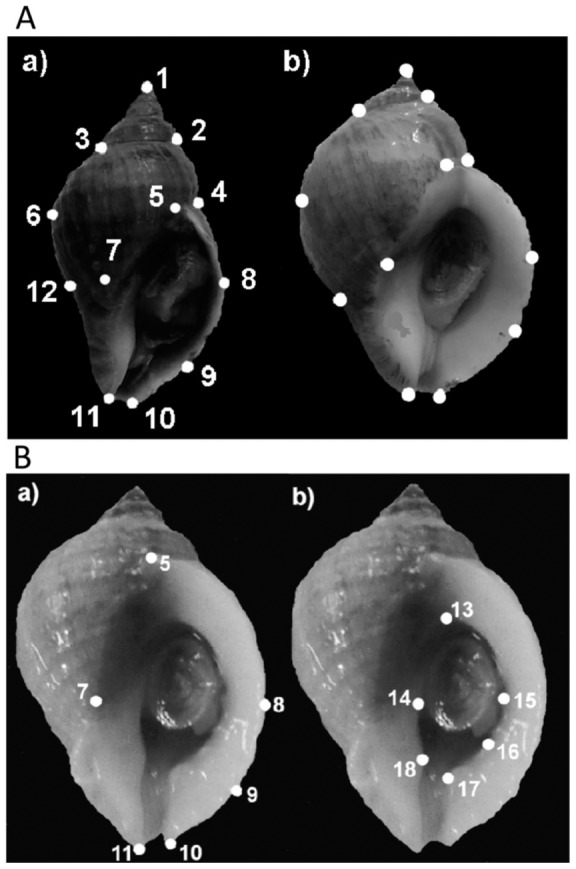
Shell shape of *Nucella lapillus*: position of landmarks. A) Shell shape a) shell collected from the site relatively sheltered from wave action, b) shell collected from the site exposed to strong wave action; shell length was represented by distance between landmarks1 and 11, shell width between landmarks 4 and 6, aperture external length between landmarks 5 and 11, aperture external width between landmarks 7 and 8, aperture internal length between landmarks 13 and 18, aperture internal width between landmarks 14 and 15; B) aperture shape a) external aperture, b) internal aperture.

Procedural details are given in Pascoal et al. [18]. Briefly, juveniles 8–12 mm in shell length were labelled with a waterproof pen according to provenance (exposed or sheltered site) and treatment (transplant or control). In August 2008, snails were transplanted from the exposed- to the sheltered-site and vice versa while control snails were replanted at their native sites. In September 2009 marked individuals were collected, photographed and retained in the laboratory. tpsDig [23], [24] was used to place landmarks on photographic images (Fig. 1) and MODICOS [25] was used to derive absolute measures of length and width (Fig. 1). Shell-lip thickness was measured to 0.01 mm using digital callipers at three points along the lip-margin while avoiding any labial teeth [4].

### Common garden experiment

A ‘control garden’ and a ‘treatment garden’, each with two replicates, were established in four 25-litre aquaria. The control garden presented an artificial environment lacking most natural characteristics including the effects of wave exposure typical of the exposed field site and of crab predation characterizing the sheltered field site. The treatment garden presented an olfactory cue signaling risk of crab predation. Seawater was supplied via two constant-head cisterns at a rate of 3 ml s^−1^with permanent aeration supplied from air-diffusion stones. Ambient temperature fluctuated seasonally between 8–16°C. Twenty-five F_1_s of sheltered site ancestry and 90 of exposed site ancestry were allocated per tank (fewer juveniles of sheltered site ancestry were obtained from the brood stock, causing imbalance in numbers per treatment). After allowing snails to acclimatize for 1 wk, two tanks were left unchanged as controls and two were supplied with water-borne olfactory cues assumed to be perceived by *N. lapillus* as risk of crab predation [26]. To generate the olfactory cues, four *Carcinus maenas*, carapace width 8–12 cm, collected from the Menai Strait, were placed in the 25-litre cistern supplying the treatment-tanks and fed cracked adult *N. lapillus*. Crabs that died were replaced within 48 h. Tanks were spatially transposed at monthly intervals to avoid incidental position effects. Barnacles attached to stones were renewed as needed to maintain an unlimited supply of food for the *N. lapillus* in each tank.

At 6 months and again at 12 months from the beginning of the experiment, snails were photographed and their shell-lip thickness measured as above. Shell length and width were measured using tpsDig and MODICOS software, as above.

At 12 months, tissue and shell mass were measured (±0.0001 g) after breaking the shell, extracting the tissue and drying for 24 h at 80°C. This procedure could be applied only to snails of exposed-site ancestry, as destructive sampling for a parallel study had depleted the sheltered-site lineage.

The F_1_ generation fortuitously laid eggs in all tanks. Hatchlings (F_2_s) were grown for 10 months, then genetically assigned to exposed- or sheltered-site ancestry (for details see Pascoal et al. [18]) and subjected to measurement as above. Again, tissue and shell mass were measured only for the exposed-site lineage, as too few snails of sheltered-site ancestry were represented by the F_2_ generation.

### Statistical analysis

Mean shell-lip thickness, shell mass and tissue mass were adjusted to appropriate covariates by ANCOVA of log_10_-transformed data. Heterogeneity of slopes was always non-significant (Treatment*Covariate P>0.05) and comparison of adjusted means was based on models lacking the interaction term.

Shape of the shell and of the external and internal rims of the aperture was quantified by using tpsRelwto generate relative warps. After confirming their independence of centroid size, scores on the first three relative warps were subjected to MANOVA (SPSS 11.3). To aid interpretation of relative warp analysis, width adjusted to length was compared among treatments by ANCOVA of log_10_-transformed data.

### Permissions

No specific permits were required for the described field studies, which took place on shores with public right-of-way and did not involve endangered or protected species. The location is not privately-owned or protected in any way.

## Results

### Reciprocal transplant experiment

**Figure 2:**
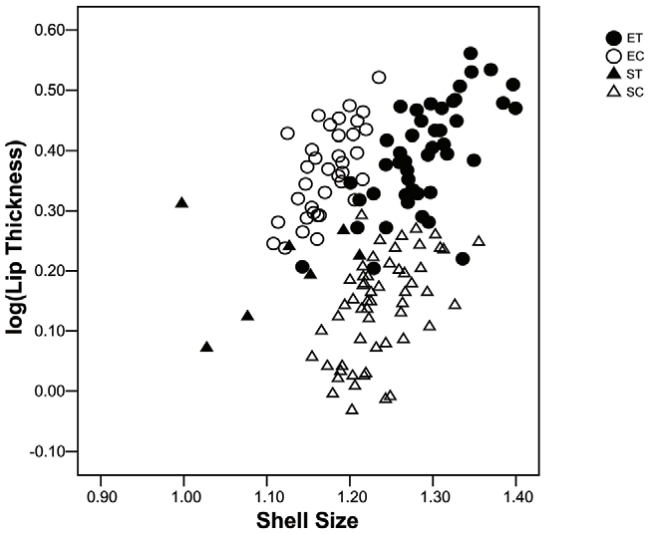
Thickness of the shell lip after 12 months growth in the field, plotted as a function of shell size (**geometric mean of width and length**)**.** ET  =  exposed-site snails transplanted to the sheltered site (n = 46), EC  =  exposed-site snails replanted at the exposed site (n = 36), ST  =  sheltered-site snails transplanted to the exposed site (n = 7), SC  =  sheltered-site snails replanted at the sheltered site (n = 56).

Statistical comparison of mean log(shell-lip thickness) adjusted to the geometric mean of shell length and width, hereafter referred to as shell size, between transplants and controls within lineages was invalidated by disparate covariate values (Fig. 2); other comparisons were unaffected. Relative shell-lip thickness was similar between snails transplanted to the sheltered site and those transplanted to the exposed site, greater in controls remaining at the exposed site than in transplants to the exposed site, but less in controls remaining at the sheltered site than transplants to the sheltered site (ANCOVA: Levene's test, P = 0.395, Bonferroni-corrected post-hoc comparisons P<0.001, except ST v. EC, P = 0.006 and ET v. ST, P>0.999. Back-transformed means with standard errors: ET  = 2.15±0.06 mm, EC  = 2.67±0.08 mm, ST  = 2.12±0.14 mm, SC  = 1.36±0.03 mm, adjusted to shell size 17.03 mm).

Unadjusted mean log(shell-lip thickness) was similar between controls and transplants within each lineage (ANOVA, Levene's test, P = 0.670, Bonferroni-corrected post-hoc comparisons: ET v. EC, P>0.999, ST v. SC, P = 0.319, all other comparisons P<0.001).

### Common garden experiment

#### Foraging behaviour and reproductive output

Within 12h of exposure to crab-effluent, snails tended to crawl up the sides of the tanks and were less frequently seen than controls among stones bearing the food supply of barnacles. Snails receiving crab-effluent produced fewer egg capsules than controls (means and standard errors: crab-effluent 54±8.0, control 247.5±27.5; t = 6.75, P = 0.021), but there was no difference in the size of egg capsules produced (crab-effluent 6.7±0.2 mm, control 7.1±0.1 mm, t = 1.56, P = 0.126) or in the number of eggs per capsule (crab-effluent 14.4±3.7; control 12.1±1.9; t = 1.90, P = 0.198).

#### Growth in shell length

**Figure 3:**
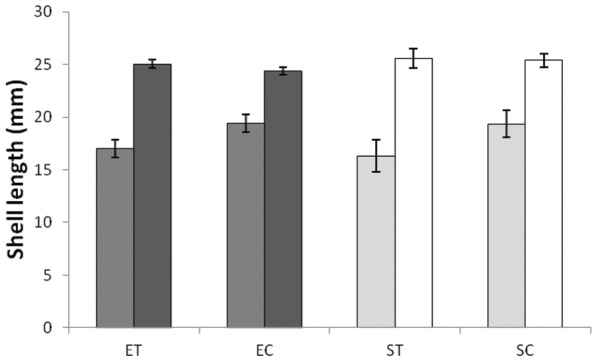
Common garden experiment: Shell length of F_1_ snails after 6 and 12 months growth. ET  =  snails of exposed-site ancestry subjected to crab-effluent (6 mo, N = 13; 12 mo, N = 38), EC  =  control snails of exposed-site ancestry (6 mo, N = 14; 12 mo, N = 51), ST  =  snails of sheltered-site ancestry subjected to crab-predation odour (6 mo, N = 4; 12 mo, N = 8), SC  =  control snails of sheltered-site ancestry (6 mo, N = 6; 12 mo, N = 16).

At 6 months from the beginning of the experiment (F_1_ generation) mean shell length was ranked higher in controls than in snails receiving crab-effluent (Fig. 3), but the contrast was statistically significant only for sheltered-site snails (ANOVA, Levene's test, P = 0.029, Treatment F_3,33_ = 2.159, P = 0.112, planned comparisons ST v. SC, P = 0.132; ET v. EC, P = 0.051). At 12 months from the beginning of the experiment, mean shell length was not significantly different among categories (Fig. 3, ANOVA, Levene's test, P = 0.132, Treatment F_3,109_ = 0.909, P = 0.439), whereas in the F_2_ generation (exposed-site only) mean shell length was greater for control than for treatment snails (treatment 19.1±0.5 mm, control 17.8±0.4 mm, t = 2.079, P = 0.043).

#### Growth in shell and tissue mass of exposed-site snails

**Figure 4:**
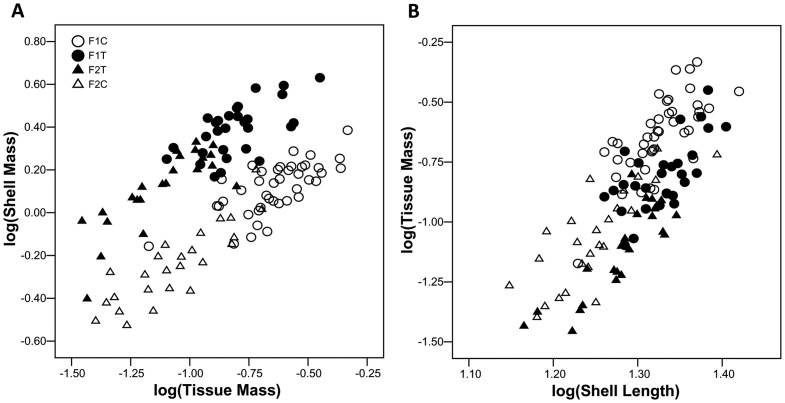
Common garden experiment, exposed-site snails at 12 months from the beginning of the experiment. (A) shell mass plotted as a function of dry tissue mass. (B) tissue mass plotted as a function of shell length. F1T  =  F_1_ snails subjected to crab-effluent (n = 29), F1C  =  F_1_ control snails (n = 41), F2T  =  F_2_ snails subjected to crab-effluent (n = 23), F2C  =  F_2_ control snails (n = 25).

Log(shell mass) adjusted to log(tissue mass) was greater in snails exposed to crab-effluent than in controls, but within those categories there was no difference between generations (Fig. 4A, Table 1). Log(tissue mass) adjusted to log(shell length) of snails exposed to crab-effluent was less than that of controls and within both of those categories was less in F_2_ than in F_1_ snails (Fig. 4B, Table 1).

**Table 1:** Shell and tissue mass at 12 months from the beginning of the experiment.

Shell	F_1_C	F_2_T	F_2_C	Mean (g)
F_1_T	**<0.001**	0.130	**<0.001**	2.244^ +0.094^_−0.091_
F_1_C		**<0.001**	0.070	0.933^ +0.042^_−0.041_
F_2_T			**<0.001**	1.892^ +0.108^_−0.102_
F_2_C				0.767^ +0.040^_−0.038_

**Tissue**	F_1_C	F_2_T	F_2_C	
F_1_T	**<0.001**	**<0.001**	>.0999	0.128^ +0.007^_−0.006_
F_1_C		**<0.001**	**<0.001**	0.194^ +0.008^_−0.008_
F_2_T			**<0.001**	0.088^ +0.005^_−0.005_
F_2_C				0.125^ +0.008^_−0.007_

Log(shell mass) adjusted to log(dry tissue mass): ANCOVA, Levene's test, P = 0.934, means adjusted to back-transformed covariate value of 0.137 g. Log(tissue mass) adjusted to log(shell length): ANCOVA, Levene's test, P = 0.775, means adjusted to back-transformed covariate value of 20.0 mm. Probabilities for Bonferroni-corrected post-hoc comparisons and back-transformed adjusted means and standard errors are tabulated. F_1_T  =  snails of the F_1_ generation receiving the crab-effluent treatment, F_1_C  =  F_1_ controls, F_2_T  =  snails of the F_2_ generation receiving the crab-effluent treatment, F_2_C  =  F_2_ controls.

#### Shell-lip thickness

**Figure 5:**
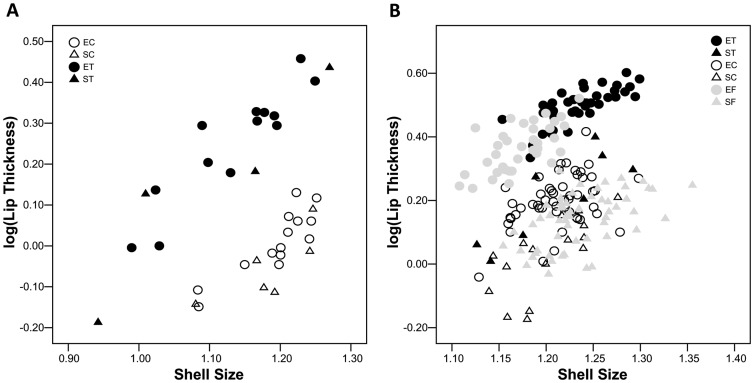
Common garden experiment: shell-lip thickness plotted as a function of shell size (**geometric mean of length and width**)**,** (**A**) **at 6 months from the beginning of the experiment,** (**B**) **at 12 months.** EF  =  exposed-site controls snails (n = 36), SF  =  sheltered-site control snails (n = 56), other symbols and sample sizes as in Fig. 2.

At 6 months from the beginning of the experiment, log(shell-lip thickness) adjusted to shell size was greater in snails receiving crab-effluent than in controls, but within those categories there was no difference between lineages (Fig. 5A, Table 2). At 12 months from the beginning of the experiment (Fig. 5B, Table 3), shell-lip thickness was greater by 85.4% in exposed-site snails experimentally exposed to crab-effluent than in controls and by 48.7% in sheltered-site snails. Control shells of both lineages had thinner shell lips than samples taken from the field at the end of the experiment, the difference being much more pronounced in the exposed-site lineage. Shell-lip thickness of snails receiving crab-effluent exceeded that of field samples in both lineages.

**Table 2:** Shell-lip thickness at 6 months.

Category	EC	ST	SC
ET	**<0.001**	0.637	**<0.001**
EC		**<0.001**	0.675
ST			**<0.001**

ANCOVA was used to adjust log(shell-lip thickness) to size expressed as the geometric mean of length and width of the shell. Probabilities for Bonferroni-corrected post-hoc comparisons are tabulated. Levene's test, P = 0.038. Back-transformed means (adjusted to shell size 14.5 mm) and standard errors: ET 1.95 ^+0.08^_−0.08_ mm, EC 0.911 ^+0.04^_−0.11_ mm, ST 1.72 ^+0.12^_−0.11_ mm, SC 0.82 ^+0.05^_−0.05_ mm. Labels are as in Table 1.

**Table 3:** Shell-lip thickness at 12 months.

Category	ET	EC	SF	ST	SC
EF	0.057	**<0.001**	**<0.001**	**<0.001**	**<0.001**
ET		**<0.001**	**<0.001**	**<0.001**	**<0.001**
EC			**<0.001**	>0.999	**<0.001**
SF				**<0.001**	**0.014**
ST					**<0.001**

ANCOVA as in Table 4, Levene's test, P = 0.007. Back-transformed means (adjusted to shell size 16.4 mm) and standard errors: EF 2.64 ^+0.08^_−0.08_ mm, ET 2.96 ^+0.09^_−0.09_ mm, EC 1.59 ^+0.03^_−0.03_ mm, SF 1.29 ^+0.03^_−0.03_ mm, ST 1.65 ^+0.10^_−0.09_ mm, SC 1.11 ^+0.04^_−0.04_ mm. EF  =  exposed-site field sample, SF  =  sheltered-site field sample.

#### Shell morphology

**Figure 6:**
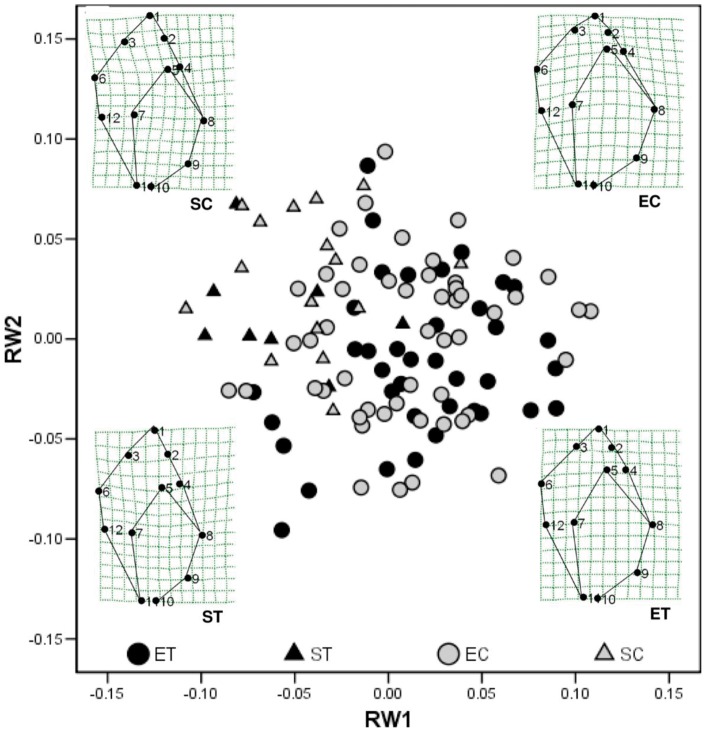
Common garden experiment: ordination of scores on the first and second relative warps produced by morphometric analysis. Symbols and sample sizes as in Fig. 2. Deformation grids correspond to individuals subjectively chosen to represent the central tendency in each treatment.

*Shape of the shell* Mean scores on RW3 differed between treatment and control snails of exposed-site ancestry, all other comparisons being statistically non-significant (Fig. 6, Table 4). Relative width (ANCOVA) was greater in shells of exposed-site snails receiving crab-effluent than in controls, but there was no difference between treatment and control shells of sheltered-site snails (Table 5).

**Table 4:** Morphometric analysis of shape of the shell, aperture external rim and aperture internal rim.

	RW1	RW2	RW3
**Shell shape**			
Variance explained	30%	22%	10%
ET v. EC	0.786	0.063	**<0.001**
ST v. SC	0.390	0.229	0.429
**Ap. external rim**			
Variance explained	42%	21%	13%
ET v. EC	**<0.001**	0.384	**<0.001**
ST v. SC	0.462	0.799	0.511
**Ap. internal rim**			
Variance explained	27%	18%	16%
ET v. EC	**<0.001**	0.104	**0.002**
ST v. SC	0.059	0.633	0.173

Probability values for planned comparisons of mean relative warp scores are tabulated. ET  =  exposed-site snails receiving crab-effluent (n = 39), EC  =  exposed-site control (n = 51), ST  =  sheltered-site snails receiving crab-effluent (8), SC  =  sheltered-site controls (n = 16).

Shell shape: MANOVA, Box's M: P = 0.097; Wilk's lambda, P<0.001; Levene's Test: RW1, P = 0.515, RW2, P = 0.386; RW3, P = 0.002.

Aperture external rim: MANOVA, Box's M: P = 0.004; Levene's Test: RW1, P = 0.235, RW2, P = 0.562; RW3, P = 0.233.

Aperture internal rim: MANOVA, Box's M: P = 0.050; Levene's Test: RW1, P = 0.321, RW2, P = 0.404; RW3, P = 0.256.

**Table 5:** Relative width of the shell, aperture external rim and aperture internal rim.

	Shell width	Ap. external rim	Ap. internal rim
**Comparison**			
ET v. EC	**<0.001**	0.095	**<0.001**
ST v. SC	0.299	0.208	0.988
**Means and SEs mm**			
	ET 14.39^ +0.14^_−0.14_	ET 11.32^ +0.11^_−0.11_	ET 5.77 ^+0.06^_−0.06_
	EC 13.43^ +0.13^_−0.13_	EC 11.04^ +0.11^_−0.11_	EC 6.56 ^+0.07^_−0.06_
	ST 12.94^ +0.26^_−0.25_	ST 9.98^ +0.30^_−0.29_	ST 6.11 ^+0.12^_−0.12_
	SC 12.62^ +0.13^_−0.12_	SC 10.38^ +0.21^_−0.20_	SC 6.10 ^+0.18^_−0.18_
**Length mm**	24.67	18.26	9.88

ANCOVA was used to adjust log(width) to log(length) within each category. Probability values for planned comparisons of adjusted means, together with back-transformed adjusted means and standard errors are tabulated. Labels are as for Table 1. Length  =  back-transformed covariate value used to adjust means. Levene's test: shell shape, P = 0.041, aperture external rim, P = 0.954, aperture internal rim, P = 0.644.

*Shape of the aperture external rim* Mean scores on RW1 and RW3 differed between treatment and control snails of exposed-site ancestry, but not of sheltered-site ancestry (Table 4). Relative width (ANCOVA) was not influenced by treatment (Table 5).

*Shape of the aperture-internal rim* Mean scores on RW1 and RW3 differed between treatment and control snails of exposed-site ancestry, but no other comparisons were statistically significant (Table 4). Relative width (ANCOVA) in exposed-site snails receiving crab-effluent was less than that of controls, but the qualitatively similar contrast in sheltered-site snails was statistically non-significant (Table 5).

## Discussion

### Reciprocal transplant experiment

Shells of incoming transplants reached a comparable size to shells of resident controls, becoming larger at the sheltered than at the exposed site in accordance with expected foraging opportunity [18]. Correspondingly, transplants from the exposed to the sheltered site grew larger shells than controls remaining at the exposed site and transplants from the sheltered to the exposed site grew smaller shells than controls remaining at the sheltered site. Transplants from the exposed to the sheltered site developed markedly thicker shell-lips, adjusted for size, than controls resident at the sheltered site, whereas transplants from the sheltered to the exposed site developed thicker shell lips than controls remaining at the sheltered site but not as thick as controls resident at the exposed site. Differences in size-range invalidated statistical comparison of adjusted means between transplants and controls within lineages. Nevertheless it is clear from Fig. 2 that although within each lineage transplants and controls grew to different sizes, they attained similar shell-lip thickness. Such trait-constancy is potentially explicable by heritable control of variation, compensatory plasticity, or both.

### Common garden experiment

Risk of crab-predation perceived via water-borne cues partially inhibited foraging, with consequent reduction in tissue growth (Fig. 4B) and reproductive output. While fewer egg capsules were produced under risk of crab predation, the size of the capsules and the number of eggs they contained remained unaffected, suggesting that reduction in fitness caused by predation risk involves the quantity rather than quality of progeny, as might be expected if the response is simply a result of starvation.

Reduction in linear shell growth was evident at 6 months from the beginning of the experiment, but not at 12 months when shell length had approached an asymptote common to all groups (Fig. 3). Except for the lack of a treatment effect on asymptotic shell length, the above results corroborate previous studies demonstrating the negative influence of perceived risk of crab predation on growth and reproduction [5], [8], [11], [27]. Faster growing snails would approach asymptote relatively early, allowing others to catch up toward the end of the experiment, obscuring the earlier effect of starvation on growth in shell length. The common asymptote indicates a lack of heritable variation in shell length and implies that the smaller mean length of exposed-compared with sheltered-site controls recorded in the reciprocal transplant experiment was environmentally induced, probably through constrained foraging opportunity [28].

Relative shell-lip thickness of juveniles at 6 months was markedly greater in snails receiving crab-effluent than in controls. Such an early ontogenetic response will be especially effective in promote fitness because vulnerability to crab predation falls most heavily upon juveniles whose shells have not yet reached a size-refuge from predation [12]. Magnitude of response was similar between lineages, with no evidence of heritable trait-variation during the juvenile phase of development. As shape was only slightly affected by predation risk in our experiment (see below), shell mass standardized to length serves as a reliable index of overall shell thickness, which again was markedly increased by exposure to crab-effluent. Shell mass standardized to tissue mass has been used as an index of resource allocation to defense [8]. Exposure to crab-effluent caused a marked increase in shell mass per unit tissue mass in both F_1_ and F_2_ generations of exposed-site snails (there were too few F_2_ sheltered-site snails for analysis). Although the marked increase in shell mass per unit tissue mass of snails experiencing crab-effluent is consistent with proposals that increased shell thickness is an actively induced defensive response to risk of crab predation [4], [5], [8], [27], [29], [30], the same result could derive passively from differential effects of starvation on shell deposition and tissue production. Starvation itself may promote thickening because calcium carbonate deposition continues unabated while linear shell growth decelerates as a result of reduced tissue growth [5]. By controlling food supply as well as predation risk, Bourdeau [11] showed that starvation resulting from constrained foraging behaviour was sufficient to explain the predator-induced shell thickening he observed in *Nucella lamellosa*.

At 12 months, the difference in relative shell-lip thickness between snails receiving crab-effluent and controls had become much greater in the exposed-site than in the sheltered-site lineage. Experimentally induced changes in shell shape were subtle and statistically significant only for exposed-site snails. Snails exposed to crab-effluent had slightly wider shells than controls, with narrower aperture internal rims. Narrowing of the internal rim of the aperture, with little change in the external rim, reflects thickening of the rim itself. Thickening in response to crab-effluent, however, may be confounded with thickening due to sexual maturation [2]. Indeed, relative shell-lip thickness of snails receiving crab-effluent exceeded not only that of controls, but also that of field-collected snails, suggesting reinforcing effects of crab-effluent and sexual maturation. As all experimental snails were born under similar laboratory conditions, the above difference between lineages indicates heritable variation expressed in later ontogeny, probably associated with sexual maturation and perhaps explaining the occurrence of thicker shells at the exposed site.

In conclusion, our common garden experiment corroborates previous studies demonstrating a negative influence of crab-effluent on prey foraging activity, tissue growth and shell growth, and a corresponding positive influence on shell thickness. Our results are consistent with, but do not critically test, the hypothesis that shell thickening is a secondary consequence of starvation rather than a direct response to predation risk. Further support of the starvation hypothesis, however, may be adduced from the slower growth and development of thicker shells by snails transplanted from the sheltered to the exposed field-site where feeding opportunities are more frequently constrained by weather than at the sheltered site.

Whereas greater plasticity has been reported in *Littorina saxatilis* [21] and *Nucella lamellosa* [20] from habitats with higher predation risk, no such differential response was evident in our populations of *N. lapillus*, at least during the vulnerable juvenile phase. Induction of defensive phenotype might be expected to be stronger in populations from habitats where risk of predation is high when integrated over time, but which varies unpredictably in the shorter term [31]. Alternatively, it could be argued that genetic control of defensive morphology should assume greater importance where generations consistently face high predation risk, in which case a weaker plastic response would be expected than in populations from low-risk habitats. Accordingly, Palmer [8] reported lesser shell-thickening in response to crab-effluent by *N. lapillus* from a sheltered than from an exposed site, situated within 14 km of our sites. Development of secondary sexual characters such thickening of the shell rim further complicates interpretation. Moreover, even congeners may show contrasted morphological changes. Whereas *N. lapillus* develops a narrower aperture correlated with an elongated spire [1], *N. lamellosa* develops greater globosity in response to crab-effluent [20]. Clearly, present knowledge is insufficient to yield broad generalizations on population variation in the relative strengths of heritable and plastic control of defensive shell morphology, or even on the detail of morphological change itself. In this regard, studies of populations within and among taxa from a wider range of habits varying in predation risk are needed.

## References

[1] Kitching JA, Muntz L, Ebling FJ (1966) Ecology of Lough Ine. 15. Ecological significance of shell and body forms in *Nucella* . Journal of Animal Ecology 35: 113–126.

[2] Crothers JH (1985) Dog-whelks an introduction to the biology of *Nucella lapillus* . Field Studies 6: 291–360.

[3] Trussell GC, Etter RJ (2001) Integrating genetic and environmental forces that shape the evolution of geographic variation in a marine snail. Genetica 112: 321–337. 11838773

[4] Edgell TC, Rochette R (2008) Differential snail predation by an exotic crab and the geography of shell-claw covariance in the northwest Atlantic. Evolution 62: 1216–1228. 18298647 10.1111/j.1558-5646.2008.00350.x

[5] Appleton RD, Palmer AR (1988) Water-borne stimuli released by predatory crabs and damaged prey induce more predator-resistant shells in a marine gastropod. Proceedings of the National Academy of Sciences of the United States of America 85: 4387–4391. 16593946 10.1073/pnas.85.12.4387 PMC280434

[6] Sepulveda RD, Jara CG, Gallardo CS (2012) Morphological analysis of two sympatric ecotypes and predator-induced phenotypic plasticity in *Acanthina monodon* (Gastropoda: Muricidae). Journal of Molluscan Studies 78: 173–178.

[7] Moody RM, Aronson RB (2012) Predator-induced defenses in a salt-marsh gastropod. Journal of Experimental Marine Biology and Ecology 413: 78–86.

[8] Palmer AR (1990) Effect of crab effluent and scent of damaged conspecifics on feeding, growth, and shell morphology of the Atlantic dogwhelk *Nucella-Lapillus* (L). Hydrobiologia 193: 155–182.

[9] Brookes JI, Rochette R (2007) Mechanism of a plastic phenotypic response: predator-induced shell thickening in the intertidal gastropod *Littorina obtusata* . Journal of Evolutionary Biology 20: 1015–1027. 17465912 10.1111/j.1420-9101.2007.01299.x

[10] Trussell GC, Ewanchuk PJ, Bertness MD (2003) Trait-mediated effects in rocky intertidal food chains: Predator risk cues alter prey feeding rates. Ecology 84: 629–640.

[11] Bourdeau PE (2010) An inducible morphological defence is a passive by-product of behaviour in a marine snail. Proceedings of the Royal Society B-Biological Sciences 277: 455–462. 10.1098/rspb.2009.1295 PMC2842639 19846462

[12] Hughes RN, Elner RW (1979) Tactics of a predator, *Carcinus maenas*, and morphological responses of the prey, *Nucella lapillus* . Journal of Animal Ecology 48: 65–78.

[13] Vermeij GJ (1982) Phenotypic evolution in a poorly dispersing snail after arrival of a predator. Nature 299: 349–350.

[14] Seeley RH (1986) Intense natural selection caused a rapid morphological transition in a living marine snail. Proceedings of the National Academy of Sciences of the United States of America 83: 6897–6901. 16593760 10.1073/pnas.83.18.6897 PMC386617

[15] Etter RJ (1988) Asymmetrical Developmental Plasticity in an Intertidal Snail. Evolution 42: 322–334. 28567855 10.1111/j.1558-5646.1988.tb04136.x

[16] Currey JD, Hughes RN (1982) Strength of the dogwhelk *Nucella lapillus* and the winkle *Littorina littorea* from different habitats. Journal of Animal Ecology 51: 47–56.

[17] Hollander J, Butlin RK (2010) The adaptive value of phenotypic plasticity in two ecotypes of a marine gastropod. BMC Evolutionary Biology 10. 10.1186/1471-2148-10-333 PMC2984422 21029403

[18] Pascoal S, Carvalho G, Creer S, Rock J, Kawaii K, et al (2012) Plastic and heritable components of phenotypic variation in *Nucella lapillus*: an assessment using reciprocal transplant and common garden experiments. PLoS ONE 7: e30289. 22299035 10.1371/journal.pone.0030289 PMC3267715

[19] Hughes RN, O'Brien N (2001) Shore crabs are able to transfer learned handling skills to novel prey. Animal Behaviour 61: 711–714.

[20] Bourdeau PE (2012) Intraspecific trait cospecialization of constitutive and inducible morphological defences in a marine snail from habitats with different predation risk. Journal of Animal Ecology 81: 849–858. 22320427 10.1111/j.1365-2656.2012.01965.x

[21] Hollander J, Adams DC, Johannesson K (2006) Evolution of adaptation through allometric shifts in a marine snail. Evolution 60: 2490–2497. 17263111

[22] Hughes RN (1986) A Functional Biology of Marine Gastropods. Croom Helm, London & Sydney.

[23] Rohlf FJ (1998) On applications of geometric morphometrics to studies of ontogeny and phylogeny. Systematic Biology 47: 147–158. 12064235 10.1080/106351598261094

[24] Rohlf FJ, Bookstein FL (2003) Computing the uniform component of shape variation Systematic Biology. 52: 66–69. 10.1080/10635150390132759 12554441

[25] Carvajal-Rodriguez A, Rodriguez MG (2005) MODICOS: morphometric and distance computation software oriented for evolutionary studies. Online Journal of Bioinformatics 6: 34–41.

[26] Vadas RL, Burrows MT, Hughes RN (1994) Foraging strategies of dogwhelks, *Nucella lapillus* (L) – interacting effects of age, diet and chemical cues to the threat of predation. Oecologia 100: 439–450. 28306933 10.1007/BF00317866

[27] Trussell GC, Nicklin MO (2002) Cue sensitivity, inducible defense, and trade-offs in a marine snail. Ecology 83: 1635–1647.

[28] Burrows MT, Hughes RN (1989) Natural foraging behaviour of the dogwhelk, *Nucella lapillus* (Linnaeus); the weather and whether to feed. Journal of Molluscan Studies 55: 285–295.

[29] Johannesson K, RolanAlvarez E, Erlandsson J (1997) Growth rate differences between upper and lower shore ecotypes of the marine snail *Littorina saxatilis* (Olivi) (Gastropoda). Biological Journal of the Linnean Society 61: 267–279.

[30] Anderson SL, Cherr GN, Morgan SG, Vines CA, Higashi RM, et al (2006) Integrating contaminant responses in indicator saltmarsh species. Marine Environmental Research 62 Suppl: S317–321 10.1016/j.marenvres.2006.04.011 16764921

[31] Trussell GC, Matassa CM, Luttbeg B (2011) The effects of variable predation risk on foraging and growth: Less risk is not necessarily better. Ecology 92: 1799–1806. 21939076 10.1890/10-2222.1

